# Noble Metal Nanoparticles with Nanogel Coatings: Coinage Metal Thiolate-Stabilized Glutathione Hydrogel Shells

**DOI:** 10.1021/acs.jpcc.4c00433

**Published:** 2024-02-14

**Authors:** Arghyadeep Basu, Iogann Tolbatov, Alessandro Marrone, Alexander Vaskevich, Lev Chuntonov

**Affiliations:** † Schulich Faculty of Chemistry and Solid-State Institute, Technion—Israel Institute of Technology, Haifa 3200003, Israel; ‡ Department of Physics and Astronomy, University of Padova, via F. Marzolo 8, 35131 Padova, Italy; § Institute of Chemical Research of Catalonia (ICIQ), Barcelona Institute of Science and Technology, Av. Països Catalans 16, 43007 Tarragona, Spain; ∥ Dipartimento di Farmacia, Università degli Studi “G. D’Annunzio” Chieti-Pescara, Via dei Vestini, 66100 Chieti, Italy; ⊥ Department of Molecular Chemistry and Materials Science, Weizmann Institute of Science, Rehovot 7610001, Israel

## Abstract

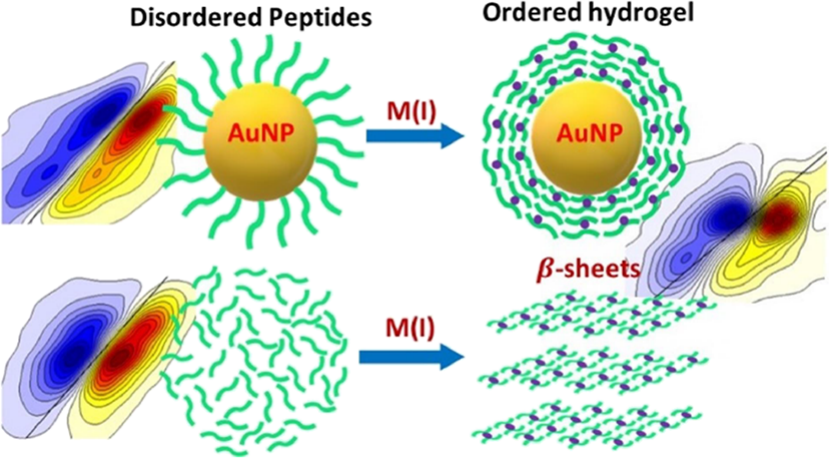

Developing biocompatible nanocoatings is crucial for biomedical applications. Noble metal colloidal nanoparticles with biomolecular shells are thought to combine diverse chemical and optothermal functionalities with biocompatibility. Herein, we present nanoparticles with peptide hydrogel shells that feature an unusual combination of properties: the metal core possesses localized plasmon resonance, whereas a few-nanometer-thick shells open opportunities to employ their soft framework for loading and scaffolding. We demonstrate this concept with gold and silver nanoparticles capped by glutathione peptides stacked into parallel β-sheets as they aggregate on the surface. A key role in the formation of the ordered structure is played by coinage metal(I) thiolates, i.e., Ag(I), Cu(I), and Au(I). The shell thickness can be controlled via the concentration of either metal ions or peptides. Theoretical modeling of the shell’s molecular structure suggests that the thiolates have a similar conformation for all the metals and that the parallel β-sheet-like structure is a kinetic product of the peptide aggregation. Using third-order nonlinear two-dimensional infrared spectroscopy, we revealed that the ordered secondary structure is similar to the bulk hydrogels of the coinage metal thiolates of glutathione, which also consist of aggregated stacked parallel β-sheets. We expect that nanoparticles with hydrogel shells will be useful additions to the nanomaterial toolbox. The present method of nanogel coating can be applied to arbitrary surfaces where the initial deposition of the seed glutathione monolayer is possible.

## Introduction

The interface between metal nanoparticles (NPs) and their environment is typically occupied by ligand molecules, whose chemical identity and structural conformation define the physical, chemical, and even optical properties of these nanomaterials.^1,2^ Noble metal NPs functionalized with peptide capping layers are thought to be biocompatible nanomaterials with controlled properties.^3^ Hydrogen bond interactions between the backbone amide units and between the side chains of the peptides shape the secondary structures of the ligands. For example, α-aminoisobutyric acid-rich peptides obtain helical conformations not only in solution but also on the surface of gold NPs (AuNPs),^4^ whereas peptides involving amino acid sequences derived from the fibril-forming proteins self-assemble into stable β-sheets.^5,6^ However, the peptide–NP surface interactions can alter the native peptide conformation and its dynamics, which can be harnessed to construct novel nanomaterials with desired functionalities.^7^

Nanogels, the mesoscopic variants of the peptide-based hydrogels, are an important class of materials involving peptide aggregates,^8^ which are extensively used in drug delivery,^9^ scaffolding for tissue engineering,^10^ and other applications.^11,12^ Nanogels can penetrate biological barriers, thus providing unique opportunities for nanomedicine and biomedical engineering research.^13,14^ Hydrogen bonding plays a central role in the structure of peptide hydrogels, and β-sheet peptide conformations are frequently found in these materials.^15^

In some cases, the hydrophobic attraction between the peptide’s side chains provides a driving force that brings together the individual strands and facilitates the formation of the hydrogen bonds between the amide units, thus initiating the nucleation of the ordered peptide aggregates.^16,17^ In other cases, metal cations mutually chelated by different peptide strands provide such aggregation-promoting interactions.^18^ On metal NPs, the peptide strands are brought together by their affinity to the surface, which can initiate aggregation.^19^ However, a complex interplay of the multitude of interactions with the surface and other ligands can also lead to inhibition of aggregation.^6,20^ Studies of amyloid-derived peptides aggregated on AuNPs revealed that the low-curvature surfaces of large AuNPs are more favorable for β-sheet formation than the high-curvature surfaces of small AuNPs.^5,6,21^ These results suggest that the self-assembly of the capping layer peptides into β-sheet aggregates requires a locally flat surface and amino acid sequences having a propensity for β-sheet formation. Indeed, in the absence of aggregation-promoting interactions, short peptides do not tend to spontaneously self-assemble into ordered aggregates, as do their longer counterparts.^7^

In the present work, we describe NP shells composed of glutathione (GSH) peptide aggregates, which bear an ordered β-sheet-like secondary structure. Within the NP shells, the peptide’s secondary structure is similar to the GSH-based hydrogel obtained by the aggregation of the peptide in the bulk solution. Therefore, noble metal NPs with ordered hydrogel shells combine the attractive properties of both NPs and nanohydrogels in a single entity.

GSH has only two amide units that can establish interstrand hydrogen bonds. Despite such a short sequence, GSH peptides obtain a β-sheet-like conformation and self-assemble into ordered intermolecular structures on the ligand-free surface of bare silver NPs (AgNPs).^22,23^ The β-sheet-like peptide assemblies stack into a multilayer molecular shell that “wraps” the AgNPs. In contrast, the naive adsorption of GSH on AuNPs does not lead to the formation of a shell of ordered peptide aggregates but rather to a disordered peptide conformation in the adsorbed monolayer.^24^ In view of the large body of literature on the amino acids’ adsorption on the noble metal surfaces, suggesting that the adsorption mechanism should not dramatically differ between Ag and Au,^25−30^ these observations indicate that the formation mechanism underlying the ordered GSH aggregates is still not understood.

In the present work, we demonstrate that the aggregation of GSH into ordered β-sheet-like aggregates on the NP surface is facilitated by M(I)–thiol coordination, where M is a coinage metal, namely, Cu, Ag, or Au. The M(I)–thiolates bridge between the cysteine side chains of the neighboring peptide strands and promote hydrogen bonding between their backbone amides. Computational studies are widely used for investigating the impact of noble metals on the aggregation of biomolecules.^31,32^ To gain insight into the molecular structure, we combined density functional computational modeling of the M(I)–GSH thiolate aggregates with two-dimensional infrared spectroscopy (2DIR) of the peptide conformation, focusing on nanogel shells on AuNPs and bulk hydrogels. We observed that the peptide aggregation mechanism on the NP surface is similar to the formation of the M(I)–thiolate-coordinated bulk hydrogels of GSH,^33−35^ which also consist of β-sheet-like peptide aggregates.

2DIR spectroscopy is a state-of-the-art spectroscopic method for the investigation of biomolecular structures.^36,37^ It provides molecular-level details by inspecting the amide-I vibrational transitions associated with the peptide’s backbone.^38,39^ When peptides obtain an ordered secondary structure, their amide groups align, and the transition dipoles produce characteristic collective modes that are sensitive to the distance and relative orientation between the amide groups. Introducing stable isotope labels on selected amino acids allows one to manipulate vibrational frequencies and extract the coupling strengths between the amide-I modes,^40^ which are then compared to those expected from the relevant structural models.^41^ 2DIR analysis of the amide-I excitons in GSH on AgNPs, which was supported by other analytical methods (localized surface plasmon resonance (LSPR), XPS (x-ray photoelectron spectroscopy), and thiol-sensitive optical probe), revealed that the aggregates are composed of stacked patches of parallel β-sheets involving ca. 8–9 peptide strands,^23^ matching the estimation of the number of GSH strands contributing to the Ag(I)–thiolate oligomers in the bulk hydrogel.^35^ Scanning electron microscopy imaging of the freeze-dried GSH hydrogel revealed that the peptides self-assemble into two-dimensional sheets,^35^ similar to what was found on the AgNP surface.^23^

Herein, we adapted the M(I)–thiolate hydrogel synthesis^33−35^ to the growth of the GSH aggregate shells on bare AuNPs while avoiding the formation of bulk aggregates. The thickness of the nanogel shell can be controlled by either GSH or the M(I) concentration, which is an important aspect for future applications. 2DIR line shapes of the amide-I transitions show that M(I)–thiolate GSH aggregates have pronounced spectroscopic signatures of the parallel β-sheet-like conformation. Ag(I)–GSH aggregates on AuNPs are more heterogeneous than Ag(I)–GSH aggregates on AgNPs, whereas Au(I)–GSH aggregates on AuNPs are the most disordered. The similarity between the molecular structure of the M(I)–GSH thiolates within NP shells and in the bulk hydrogel indicates that the characteristic size of the ordered aggregates is also similar.

## Methods

### AuNP Synthesis

#### Immobilized AuNPs (Island Films)

Gold island films (with a nominal thickness of 5 nm) were fabricated on thoroughly cleaned glass slides. Gold was evaporated at a rate of 0.1 nm s^–1^ in a cryo-HV evaporator (Key High Vacuum) equipped with a Maxtek TM-100 thickness monitor. Evaporated gold slides were then annealed at 500 °C for 10 h in a furnace (Ney Vulcan 3-550) and cooled gradually to room temperature. HRSEM (high-resolution scanning electron microscopy) images confirmed that the average dimension of gold films was 31 ± 3 nm. (See refs (42,43) for more details.)

#### Citrate-Capped AuNPs

Citrate-capped AuNPs were prepared following a modified Turkevich-Frens method.^44^ Briefly, 40 mg of HAuCl_4_-3H_2_O was added to 120 mL of boiling Millipore water (18 MOhm) and stirred well. Then, 10 mL of 40 mM hot trisodium citrate solution was added to the solution. The color of the solution turned colorless, followed by a light pinkish color, and it gradually turned red. The solution was refluxed and stirred for another 45 min and then cooled down to room temperature. TEM (transmission electron microscopy) imaging (see Figure S1 of the Supporting Information) confirmed that the size of the AuNPs was around 20 nm.

#### Bare AuNPs

Bare AuNPs were prepared by a hydrogen reduction procedure following ref (45). Briefly, 400 mg of Au_2_O_3_ (Alfa-Aesar) was added to 1 L of Millipore water (18 MOhm) in a pressure vessel (Ace Glass) equipped with a reflux condenser, heated to 90 °C, and gently stirred. The condenser was connected to a hydrogen gas (purity 99.99%). The vessel was flushed and pressurized to an excess pressure of 10 psi for 30 min. The reaction was terminated by releasing the pressure. TEM imaging indicated a particle size of ∼70 nm (see Figure S1 of the Supporting Information).

### Functionalization of AuNPs with Thiolate Shells and Preparation of Hydrogels

GSH was purchased from Acros-Organics; ^13^C-labeled GSH was synthesized by Vivitide (USA), using a protected cysteine amino acid (^13^C3 and ^15^N) from Cambridge Isotope Laboratories (USA). All other reagents were purchased from Sigma-Aldrich. All of the reagents were used as received.

Preparation of the thiolate shell on AuNPs and bulk hydrogels was achieved by the proper choice of concentrations of the corresponding solutions of metal (Ag, Cu, and Au) salt and GSH. We found that maintaining the concentration of GSH below 0.1 mM prevents the formation of bulk hydrogel. The latter were prepared by increasing the concentration of GSH to the mM region. We assume that the formation of bulk hydrogel at low GSH concentration is kinetically hindered, but a detailed analysis of the associated mechanism is beyond the scope of the current study.

#### Ag(I)–GSH Thiolates

First, 3 mL of a 1 mM AgNO_3_ solution was added to 9 mL of the bare AuNP solution. Then, 1.3 mL of 10 mM GSH was added to form an A(I)–thiolate shell on AuNPs. For hydrogel samples, 1.2 mL of a 10 mM GSH solution was added to 10 mL of a 1 mM AgNO_3_ solution. The gel formed immediately.

#### Cu(I)–GSH Thiolates

First, 40 mg of CuCl was added to 20 mL of Millipore water (18 MOhm) and was shaken very well. Since CuCl is only partially soluble in water, the supernatant was collected and passed through a syringe filter to remove the undissolved salt. Then, 3 mL of the resulting CuCl solution was added to 9 mL of bared AuNP solution. Finally, 1.3 mL of 10 mM GSH was added to form the Cu(I)–thiolate shell. For hydrogel samples, 0.5 mL of NaOH (1N) was added to 5 mL of 50 mM CuCl_2_ solution. The solution was stirred for a few minutes; then 5 mL of 100 mM GSH solution was added, and the hydrogel formed immediately.

#### Au(I)–GSH Thiolates

First, 0.1 mL of NaOH (1N) was added to 10 mL of 5 mM HAuCl_4_. Then, 3 mL of the resulting solution was added to 9 mL of bare AuNPs. Finally, 1.3 mL 10 mM GSH was added to form an Au(I)–thiolate shell. For hydrogel samples, 0.5 mL of NaOH (1N) was added to 5 mL of a 40 mM HAuCl_4_ solution. The mixture was stirred for a few minutes; then 5 mL of 100 mM GSH solution was added and the hydrogel formed immediately.

In all samples, the solvent was changed from H_2_O to D_2_O by centrifugation at 5000 rpm for 30 min, followed by the removal of the supernatant and resuspension of the pellet in D_2_O. The washing cycle was repeated three times. For the NP samples, the washing procedure also ensured that an excess of unbound GSH was removed.

### 2DIR Spectroscopy

Three mid-infrared femtosecond laser pulses were generated by the 4 kHz regenerative amplifier (Solstice Ace, Spectra Physics), followed by the OPA and DFG frequency-conversion stages (Topas, Light Conversion). The central wavelength of the excitation pulses was 6.2 μm. The pulses were split into three replicas, which were focused at the sample with BOXCAR geometry; the fourth replica pulse (local oscillator) was used for spectral interferometry to heterodyne the nonlinear signal on the 64-element liquid-nitrogen-cooled HgCdTe array detector (Infrared Systems Development) mounted on the spectrograph with the spectral resolution of ca. 7 cm^–1^. The time interval between the first and the second pulses was scanned (range of scan 3 ps) to obtain the excitation frequency axis with a spectral resolution of ca. 10 cm^–1^. All the spectra were collected with a waiting time interval between the second and the third pulses of *T* = 300 fs. Sample solutions were placed between two 2 mm-thick CaF_2_ windows with a 12 μm spacer.

### Computational Details

Density functional theory (DFT) allows for accurate characterization of transition metal complexes with biomolecules,^46−48^ including coinage metals.^49−51^ The Gaussian 16 quantum chemistry package was employed for computations; all structures were optimized with the hybrid range-corrected functional ωB97X-D^52^ and the def2SVP basis set,^53,54^ which yielded reliable geometrical structures and precise estimations of the electronic energies.^49,55^ Frequency computations verified the stationary nature of the minima and produced zero-point energy (ZPE) and vibrational corrections to the thermodynamic properties. The harmonic approximation was adopted to compute the zero-point energy and thermal and entropy corrections and yield the Gibbs free energy of each investigated system. The integral equation formalism of the polarizable comntinuum model (IEFPCM) was employed to account for the solvation free energy in water.^56^ This method yields free energies with considerably smaller errors compared to the other continuum models, both for neutral and charged complexes.^57^

## Results and Discussion

### GSH Shells on AuNPs

#### Localized Plasmon Resonance Spectroscopy

The growth of the GSH adsorbate layers on the surface of AuNP was monitored with LSPR spectroscopy: we followed the shift of the LSPR peak wavelength, Δλ, which occurs upon the changes in the effective refractive index, Δ*n*, of the NPs’ immediate environment associated with adsorption.^58^ Because colloidal NPs in solution may aggregate, providing an alternative mechanism for the appearance of the LSPR peak shift,^59^ in these experiments, we used bare AuNPs immobilized on a glass substrate, also known as metal island films (nominal film thickness of 5 nm), which provide a stable and reliable platform for tracking the growth of the peptide layer on the surface.^42,43^ The empirical relationship between Δλ and the thickness of the adsorbate layer *d* is given by Δλ = RISΔ*n* (1 – *e*^–*d*/*l*^), where RIS is the refractive index sensitivity constant, and *l* is the so-called decay length, characterizing the spatial extent of the LSPR sensitivity region.^42^

**Figure 1:**
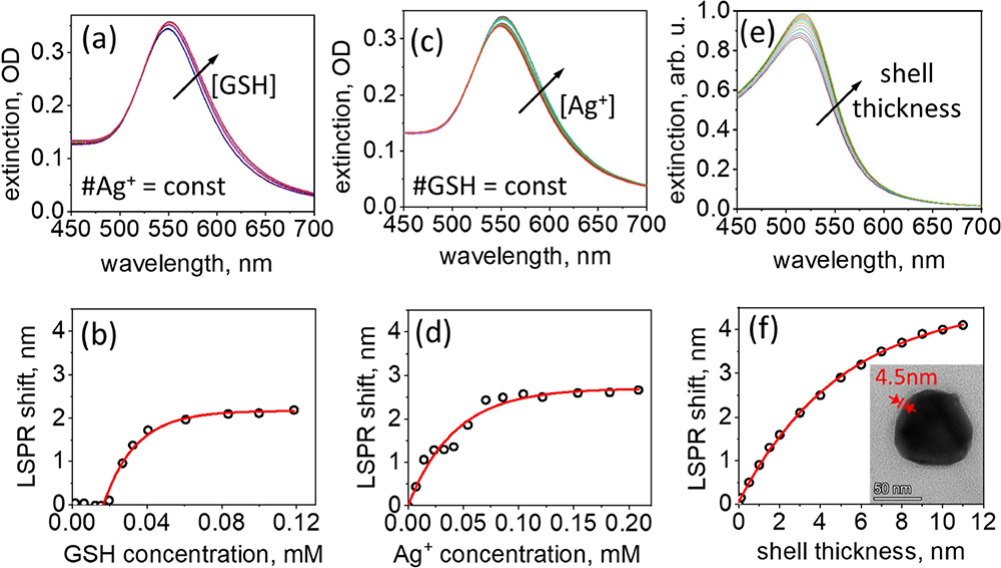
Localized plasmon resonance spectroscopy of the GSH adsorbate layer growth. Titration with GSH for a constant amount of AgNO_3_ (0.7 mM): (a) LSPR spectra and (b) LSPR shift. The lag of the LSPR shift at low concentrations suggests that a minimal GSH amount is required for the initial adsorption and growth of detectable layers of peptide aggregates. Circles denote experimental Δλ values, and red lines denote fits to the empirical expression (see the text for details). Titration with AgNO_3_ for a constant amount of GSH (0.03 mM): (c) LSPR spectra and (d) LSPR shift. (e) Extinction spectra were calculated using a dipolar polarizability model for the core–shell nanoparticle for different shell thicknesses. (f) LSPR shifts corresponding to spectra in panel e. Inset: Transmission electron microscopy of bare AuNPs with Ag(I)–GSH thiolate adsorbates. The thickness of the peptide shell is indicated in red.

A glass slide with immobilized AuNPs was immersed in 1.5 mL of 0.7 mM solution of AgNO_3_, and the sample was titrated with 10 μL portions of 0.5 mM solution of GSH. Upon addition of the titrant, the solution was agitated with the pipette and left for ca. 10 min to equilibrate. The LSPR spectra were acquired in situ with a slide dipped in solution. Sequential spectra were taken after each GSH addition without removing the slide from the cuvette and a shift of the plasmon resonance was detected. In the second type of experiment, the glass slide with AuNPs was immersed in 1.5 mL of 0.03 mM GSH solution, and the sample was titrated with 10 μL portions of 0.5 mM solution of AgNO_3_. In both scenarios, the LSPR peak, which appears in the spectrum at ∼550 nm, as shown in Figure 1, gradually shifted with the increasing amount of the added reagent and converged to Δλ ≈ 2.5 nm. Such saturation of the LSPR response indicates that either the adsorbate layer thickness exceeds the LSPR decay length and the signal is not sensitive to further changes in the environmental dielectric constant or that the growth of the GSH layer stopped, when a certain adsorbate thickness was reached. For metal island films with a nominal thickness of 5 nm, which corresponds to AuNPs of ca. 30 nm, RIS ≈ 70, whereas *l* ≈ 9 nm (see ref (42) for details). Thus, assuming a refractive index of the GSH aggregate layer of 1.4 (Δ*n* = 0.07), we found that for maximal values of Δλ, *d* ≈ 5 nm, which is well within the LSPR sensitivity range. Therefore, saturation of the plasmonic response can be ruled out.

We verified the outcome of the empirical relationship between the LSPR shift and the thickness of the NP shell using a model for the core/shell nanoparticles,^60,61^ where dipolar polarizability α is given by
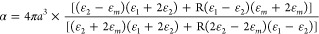
where ε_1_, ε_2_, and ε_*m*_ are the permittivities of the gold core, GSH shell, and aqueous medium, respectively, *a* is the radius of the coated nanoparticle, and *R* is the fraction of the core volume. The nanoparticle extinction cross-section, 
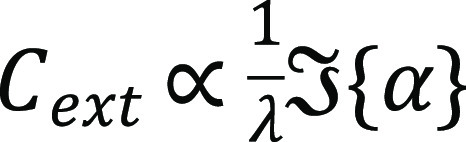
, obtained with this model, is plotted in Figure 1e, where we used the Au dielectric function from ref (62). The model predicts the LSPR wavelength of 520 nm, which is lower than in the experiment, because the point-dipole approximation used in the model does not account for the effects of nanoparticle shape,^63^ dynamic depolarization, and radiation damping,^64^ as well as for the presence of the substrate.^65^ However, this deviation does not affect our analysis, which focuses on the relative LSPR shift, Δλ. The calculated LSPR shift for various shell thicknesses in Figure 1f shows that the value of Δλ ≈ 2.5 nm (*d* ∼4.5 nm) lies within the decay length, which characterizes the LSPR sensitivity, indicating that the growth of the peptide shell can be controlled by adjusting the concentration of either GSH or M(I). These results also imply that when the concentration of both GSH and M(I) is below ca. 0.1 mM, bulk hydrogel is not formed, which allows the structural study of thiolate shell on NPs while avoiding formation of bulk hydrogel (see Methods for details). Transmission electron microscopy of the bare AuNPs with Ag(I)–GSH adsorbates confirms the formation of the uniform shell on the NP surface with a thickness of 4–5 nm, as illustrated in the inset of Figure 1f.

#### Dynamic Light Scattering Spectroscopy

**Figure 2:**
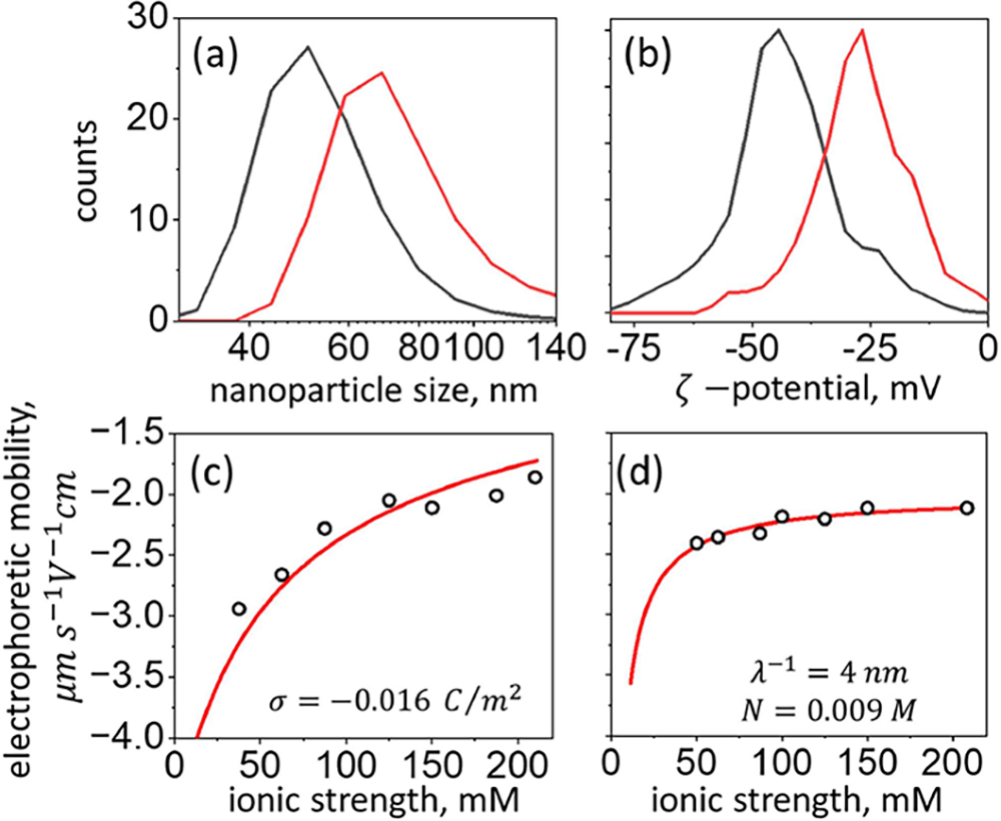
DLS spectroscopy. (a) Nanoparticle size distributions (note the logarithmic scale on the *x*-axis) and (b) ζ-potential distributions. Black lines represent bare AuNPs, and red lines represent AuNPs coated with the Ag(I)–GSH shell. Electrophoretic mobility measurements of bare AuNPs (c) and Ag(I)–GSH-coated AuNPs in phosphate buffer solution (pH 5.54). Circles represent experimental data, red lines represent fits to the models of refs (68,69) (c) and refs (70,71) (d). Surface charge density, σ, the softness parameter, λ^–1^, and the ionized group concentration within the shell, *N*, obtained from the fits are indicated.

We characterized the size distribution, the surface charge density, and the electrostatic potentials on the colloidal AuNPs using dynamic light scattering spectroscopy (DLS, Zetasizer Ultra, Malvern Instruments, U.K.). The results of the AuNP size measurements by DLS were fully consistent with the LSPR and TEM results described in the previous section. The distribution of the bare AuNPs size had a mean of 50 nm and a width of 25 nm (full width at half maximum, FWHM), and that of the AuNPs coated with the Ag(I)–GSH shell had a mean of 65 nm and a width of 33 nm, as shown in Figure 2a. We propose that the growth of the ordered thiolate layer stops when a certain density of defects in the structure is accumulated. Indeed, we were able to grow shells of the same thickness also on the surface of gold antennas^66^ having micrometer dimensions. The shell formation was observed as a redshift of the resonance frequency following the change in the dielectric constant, similar to the LSPR measurements (data not shown). Some variation in the shell thickness is expected because different crystal planes present on the surface of the multicrystalline AuNPs can accumulate the defects at a different rate. This variation is reflected in the widths of the NP size distributions obtained by DLS measurements. Note that upon coating with the thiolate shell, the width of the size distribution increased by the same factor as the distribution mean (ca. 1.3), which suggests that the coating has a similar thickness for all NPs in the ensemble. The values of the ζ-potential were −45 and −27 mV for the bare and coated AuNPs, respectively, as illustrated in Figure 2b, which confirms the good colloidal stability of both samples.^67^

The surface charge density of the bare AuNPs was obtained based on the measurements of the electrophoretic mobility, μ, in the phosphate buffer (pH 5.54) with systematically varied ionic strength. The measurements were performed in a folded capillary cell, model DTS1070 (Malvern). In order to analyze the experimental results, which are shown in Figure 2c, we followed the work of Ohshima and co-workers,^68,69^ who derived approximate expressions relating between the electrophoretic mobility, zeta-potential, ζ, and the surface charge density, σ. For the case of bare AuNPs, the model assuming the constant charge density on the surface is applicable, thus, μ = μ(σ, κ*a*).^68,69^ Here, in addition to σ, electrophoretic mobility depends on the Debye parameter (κ) and radius of the spherical NP *a*. Debye parameter is given by , where *n*^∞^ is the bulk number concentration of the symmetrical electrolyte solution with ions of valence *z*, *e* is the electron charge, ε_r_ is the relative permittivity of the solvent, ε_0_ is the vacuum permittivity, *k*_B_ is the Boltzmann constant, and *T* is the temperature. Because the theoretical expressions for μ(σ, κ*a*) are quite cumbersome, we do not replicate them here, but in the Supporting Information (see eqs S1–S3) and refer the reader to the original work of Ohshima and co-workers.^68,69^ Fitting the data to Ohshima’s equation resulted in the value of σ = −0.016 C/m^2^, which is similar to that obtained in ref (68) for the citrate-capped AuNPs.

Measurements of the electrophoretic mobility of AuNPs coated with the Ag(I)–GSH thiolate shell, as shown in Figure 2d, were analyzed with Ohshima’s model for NPs with hard core and soft permeable shell.^70,71^ Unlike in the previous case of rigid NPs, here, μ = μ(κ, *ZN*, λ^–1^), where *Z* is the valence and *N* is the concentration of the fixed ions within the soft permeable shell, and λ^–1^ is the so-called softness parameter, which characterizes the amount of friction exerted on the electrolyte flow into the shell and has units of length. For rigid NPs (i.e., without permeable shell), λ^–1^ approaches zero, whereas for soft NPs, the nonzero values of λ^–1^ indicate that the shell is swollen.^70^ Fitting the experimental data to the expressions for μ from ref (70) (see also eqs S4–S7 in the Supporting Information) results in values of *N* = 9 mM and λ^–1^ = 4 nm, which further confirm the soft character of the Ag(I)–GSH thiolate shell.

### Computational Predictions

**Figure 3:**
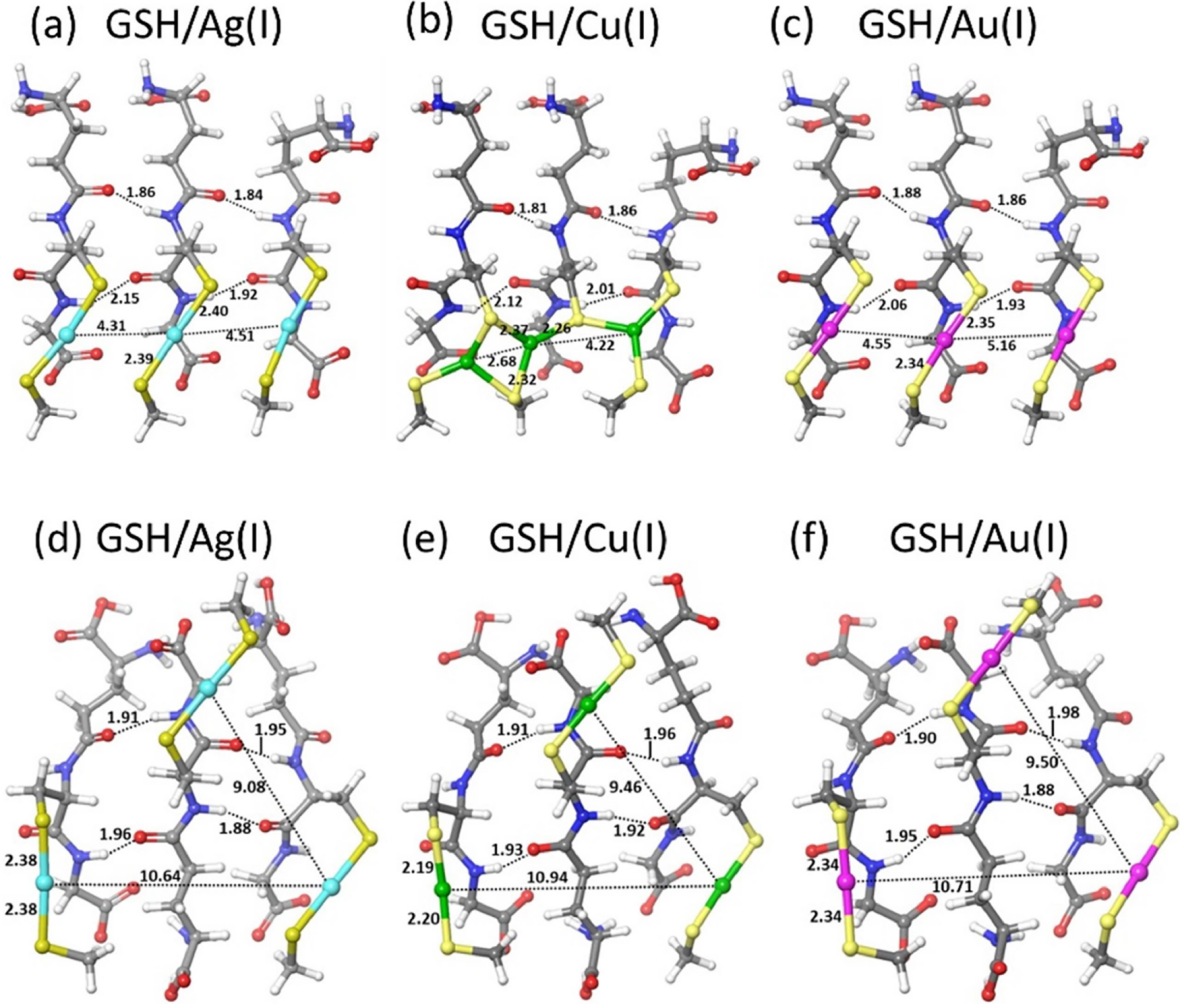
DFT-optimized structures of M(I)–thiolate GSH trimers. Top row, parallel strand orientation; bottom row, antiparallel strand orientation. Left column: M = Ag, center column: M = Cu, and right column: M = Au. The interatomic distances are indicated in angstroms. Color scheme: Ag (cyan), Cu (green), Au (magenta), S (yellow), O (red), N(blue), C (gray), and H (white).

The possibility of forming the β-sheet-like GSH aggregates in the presence of the M(I) cations of coinage metals was theoretically examined by density functional theory. Simplified models of the (GS–M(I)–SG)_*n*_ oligomers were constructed, where *n* = 3, and one of the GSH units was replaced by methanethiol, CH_3_SH. Such simplified models include what we believe to be the most important structural motif of the ordered GSH aggregates, namely, the M(I)–thiolate coordination and the interstrand hydrogen bonding of the amide groups; however, they are still small enough to be computationally tractable. Trimeric structures (GS–M(I)–SCH_3_)_3_ were optimized assuming either a parallel or antiparallel orientation of the GSH strands, and the β-sheet-like conformations were observed in both cases, as evidenced by the corresponding dihedral angles of the cysteine backbone (−126° ≤ φ_par_ ≤ – 147°; + 127° ≤ ψ_par_ ≤ + 144°; – 150° ≤ φ_anti–par_ ≤ – 134°; + 115° ≤ ψ_anti–par_ ≤ + 131°) and the NH–OC hydrogen bond lengths between the amide units (1.88–1.98 Å).^72^ The optimized structures are shown in Figure 3.

Analysis of the optimized structures reveals that for the parallel strand orientation, the S–M(I)–S coordination is linear for the Ag and Au centers (see Figure 3a, c), with M(I)–S bonds of 2.39–2.40 and 2.34–2.35 Å, respectively, and M(I)–M(I) distances of 4.31 and 4.51 Å for Ag and 4.55 and 5.16 Å for Au. The calculated distances for Ag(I) thiolates are consistent with typical experimental values, supporting the validity of our model.^73^ The Cu(I) center adopts a trigonal coordination with the electron-donating S atoms of thiols, as shown in Figure 3b, with shorter Cu(I)–Cu(I) distances of 2.68 and 4.22 Å. The higher coordination number of Cu(I) centers is not surprising because of the numerous evidence of various S-bridged complexes of Cu(I).^74^ For the antiparallel strand orientation, all M(I) centers are linearly coordinated by the GSH and CH_3_SH molecules with M(I)–S bond lengths of 2.38, 2.34, and 2.19–2.20 Å for Ag, Au, and Cu, respectively, and with the M(I)–M(I) distances lying in the narrow range of 9–11 Å, as shown in Figure 3d–f.

The calculated values of the Gibbs free energy for the antiparallel trimer structures were lower than those for their parallel counterparts, suggesting that this structural motif is more thermodynamically stable. As shown in Table 1, the energy difference between the parallel and antiparallel structures was in the range of 15–20 kcal/mol, which approximately corresponds to the energy gain of one hydrogen bond interaction. However, our energy estimates only accounted for the lateral assembly of GSH scaffolds and neglected any contribution by the mutual interaction of GSH termini; consequently, the overall stability of the antiparallel construct compared with the parallel construct cannot be definitively assessed. Our calculations show that parallel and antiparallel strand configurations lead to different periodicities of the M(I) atom arrays with higher or lower mutual proximity, respectively. For the GSH aggregates formed on the NP surface, the peptide strands may, in principle, coordinate at the oxidized metal *loci*, i.e., the M(I) centers, to form a monolayer. The lattice constant of Au is 4.08 Å, and the distances between the closest atoms on the surface are typically 2.8–2.9 Å, depending on the crystallographic plane^75^; therefore, the M(I)–M(I) distances in peptide trimers that we obtained are relatively close to the integer multiples of the relevant interatomic distances on the surface. Thus, concerning the formation of the seed GSH monolayers on the NP surface, the theoretical analysis suggests that geometrically, both parallel and antiparallel conformations can form.

**Table 1:** Gibbs Free Energies of Optimized Trimeric Structures

trimeric structures	relative GFE, kcal/mol
Ag, antiparallel	0.0
Ag, parallel	14.7
Cu, antiparallel	0.0
Cu, parallel	18.4
Au, antiparallel	0.0
Au, parallel	20.0

### 2DIR Spectroscopy

**Figure 4:**
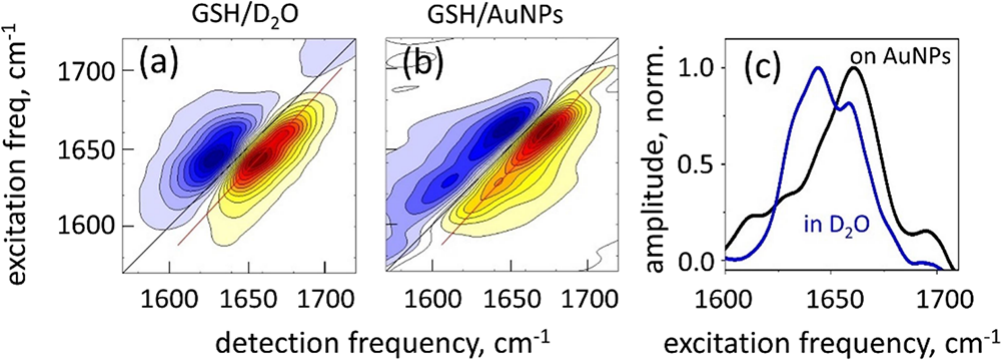
2DIR spectroscopy of disordered GSH. (a) GSH solution in D_2_O. (b) GSH self-assembled on citrate-capped AuNPs by replacing the citrate ligands with the thiol group of GSH. (c) Normalized diagonal slice amplitudes. The data are extracted along the red line of the 2DIR data (GSB/SE peaks) and projected on the vertical axis. Blue line represents panel a, and black line represents panel b.

**Figure 5:**
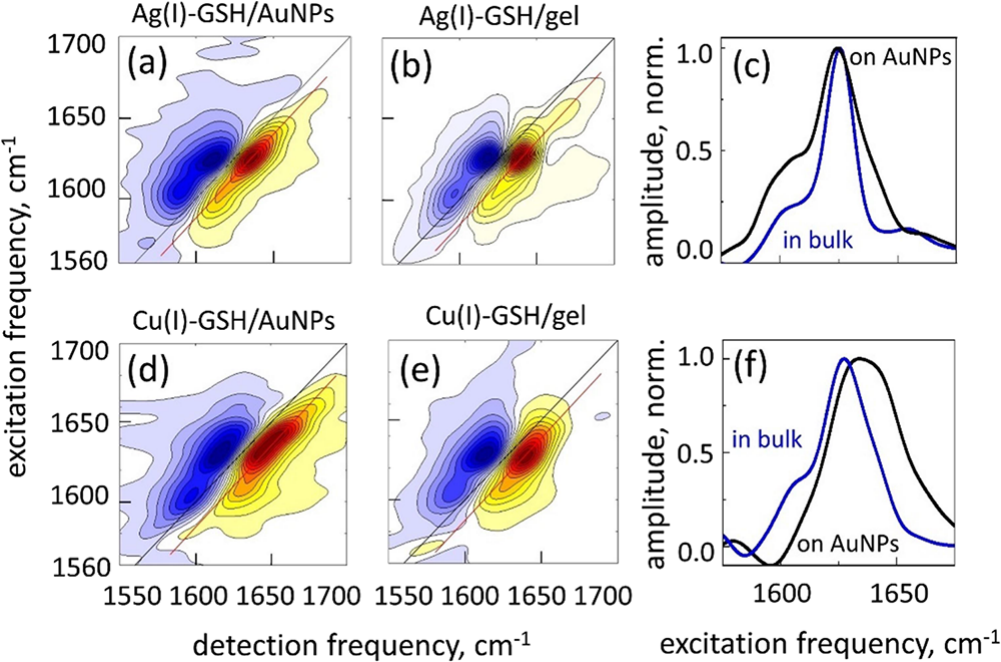
2DIR spectroscopy of GSH thiolates. Ag(I)–GSH aggregates on the surface of AuNPs (a) and in the bulk hydrogel (b). Cu(I)–GSH aggregates on the surface of AuNPs (d) and in the bulk hydrogel (e). Normalized diagonal slice amplitudes (c) and (f). The data are extracted along the red line of the 2DIR data (GSB/SE peaks) and projected on the vertical axis. Blue lines represent bulk hydrogels, black lines represent the AuNPs surface.

**Figure 6:**
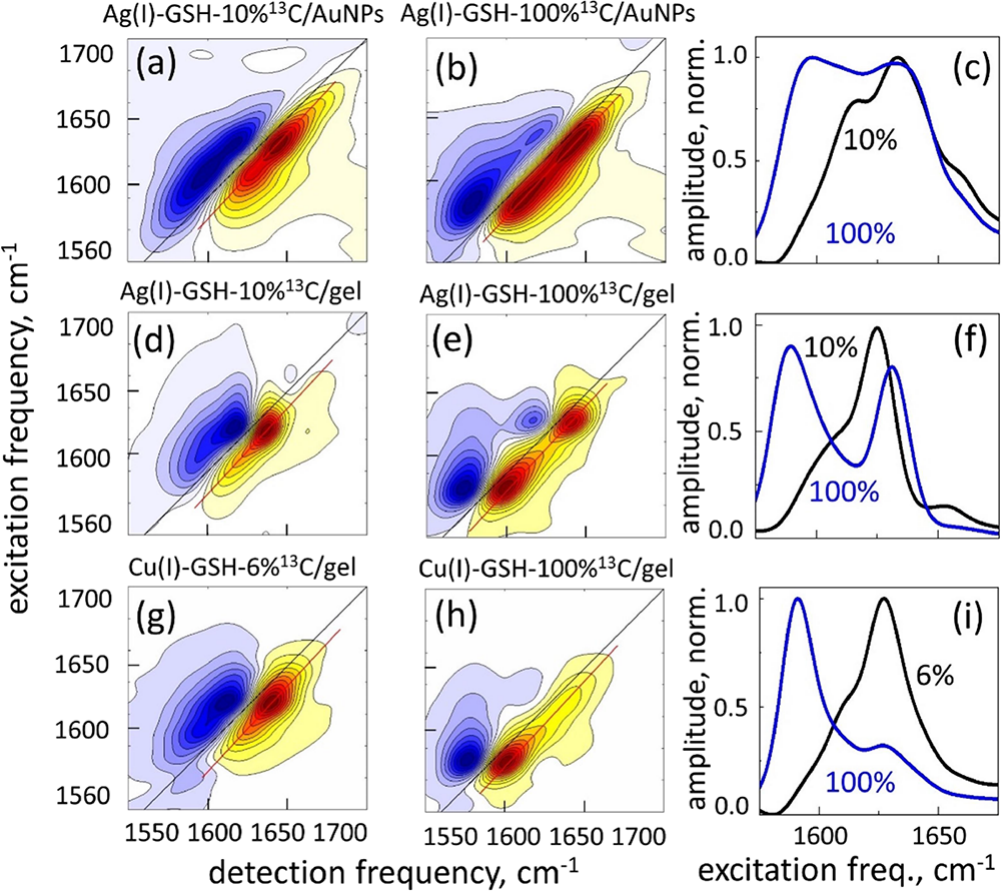
2DIR spectroscopy of the^13^C-isotope-labeled GSH thiolates. (a, b) Ag(I)–GSH on AuNPs, (d, e) Ag(I)–GSH bulk hydrogel, (g, h) Cu(I)–GSH bulk hydrogel. Left column: a low label concentration and middle column: fully labeled samples. Normalized diagonal slice amplitudes (c), (f), and (i). The data are extracted along the red line of the 2DIR data (GSB/SE peaks) and projected on the vertical axis. Black lines: low-concentration of ^13^C labels and blue lines: fully labeled samples.

**Figure 7:**
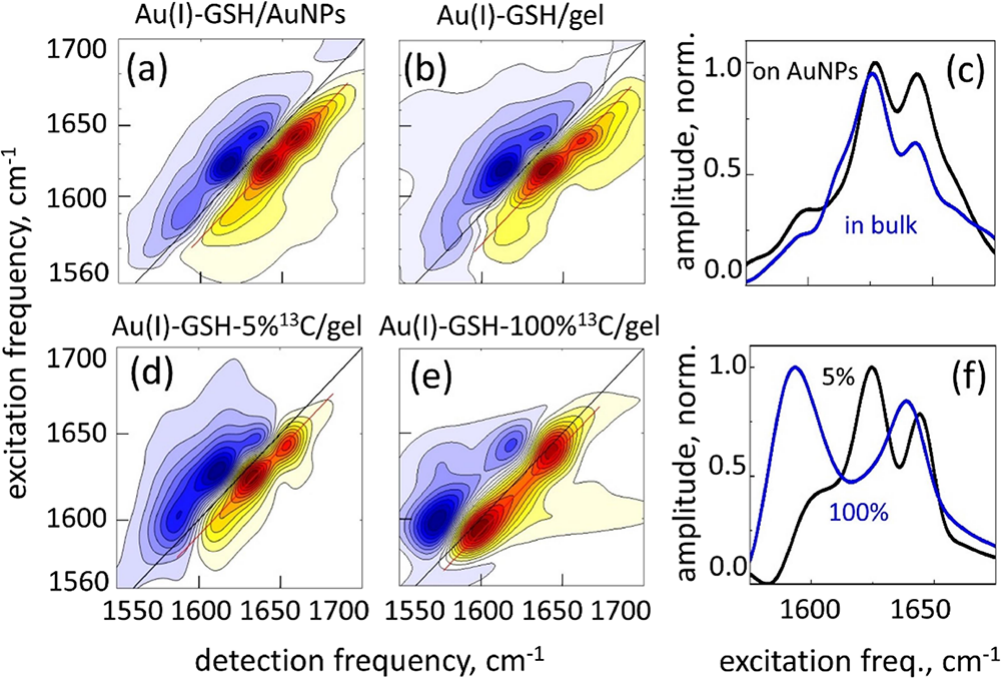
2DIR spectroscopy of Au(I) GSH thiolates. (a) Au(I)–thiolate on the surface of AuNPs, Au(I) thiolate in the bulk hydrogel: (b) unlabeled peptide; (d) low cysteine ^13^C label concentration; and (e) fully labeled peptide. Normalized diagonal slice amplitudes (c) and (f). The data are extracted along the red line of the 2DIR data (GSB/SE peaks) and projected on the vertical axis. Black lines: low concentration of ^13^C labels and blue lines: fully labeled samples.

Infrared spectroscopy is a popular method used for biomolecular structure characterization by the analysis of amide-I vibrational transitions. However, frequently, linear Fourier-transmorm infrared spectroscopy (FTIR) suffers from spectral congestion, an inability to distinguish between the homogeneous and inhomogeneous components of the transition line shape, and weak signals from the chromophores of interest being overwhelmed by the background of the environment. Third-order nonlinear 2DIR spectroscopy, conducted with sequences of ultrafast laser pulses, solves many of these problems: the nonlinear signals, which scale with the fourth power of the transition dipole moment (compared to the second power in linear spectroscopy) are better differentiated against the weak transitions contributing to the background, whereas the data is spread in two dimensions to reduce spectral congestion.^36,37^ The 2DIR spectrum (see Figures 4–7 below) is essentially a correlation map between the excitation (vertical axis) and detection (horizontal axis) frequencies accessed by the excitation pulses in the sample.^38^ Therefore, the elongation of the spectral peaks along the diagonal line of the spectrum reflects the inhomogeneous component of the transition line shape, whereas the antidiagonal width of the peak is the homogeneous component. Because the 2DIR spectrum involves nonlinear transitions, in the purely absorptive spectrum,^76^ the spectral peaks are observed in pairs having opposite signs. Below, we adopt notation, where the peaks corresponding to the fundamental transitions (ground to the first excited state) and associated with the pathways of the ground state bleach (GSB) and stimulated emission (SE) appear positive (red), whereas the overtone transitions (first to the second excited state) associated with the pathways of the excited state absorption (ESA) appear negative (blue).

### Disordered GSH Aggregates on AuNPs

In the absence of M(I) cations, the GSH self-assembles on the AuNP surface as a disordered ensemble, as evidenced by the 2DIR spectroscopy of the amide-I vibrational transitions, whose frequency in proteins and peptides is highly sensitive to the molecular conformation and the environment. Specific values of the amide-I frequency can be expanded into a zero-order term, which represents the transition frequency of the basic amide unit (e.g., in vacuum), and the higher-order terms that account for the specific peptide conformation (e.g., the dihedral angles of the backbone), for the electrostatic potential of the solvent and the surrounding amino acid residues, as well as for the transition dipole coupling to the neighboring amide-I modes.^41^ The amide-I transitions in the GSH were recently assigned using the ^13^C stable isotope labeling of the carbonyl group on the cysteine.^23^ In the acidic D_2_O solution, where we assume that the peptide is found in a disordered (random coil) conformation, the cysteine amide-I vibrational mode absorbs at ω_C_ = 1661 cm^–1^, whereas that of γ-glutamyl absorbs at ω_E_ = 1644 cm^–1^, as seen in the 2DIR spectrum in Figure 4a. Both peaks have a similar transition strength; however, the ω_E_ peak amplitude is ∼20% higher as seen in the diagonal slice of the 2DIR spectrum as shown in Figure 4c.

When GSH is added to a colloidal solution of citrate-capped colloidal AuNPs, GSH self-assembles on the surface of AuNPs via the ligand exchange of citrate for thiol. In the corresponding 2DIR spectrum shown in Figure 4b, the high-frequency amide-I peak appears three times stronger than the low-frequency one (see diagonal slice of the 2DIR spectrum in Figure 4c), with the corresponding transitions at ω_high_ = 1661 and ω_low_ = 1628 cm^–1^. The relative weakening and the redshift of ω_low_ can occur following the scenario of thiolate coordination at the atomic centers on the surface, as predicted by theoretical analysis and the consequent aggregation of the peptides. Another possibility is that the GSH monolayer attains a previously suggested conformation,^28,29,77^ where the transition dipole of the γ-glutamyl amide-I mode is oriented at a small angle to the metal surface such that the interaction with the image dipole redshifts its frequency, whereas the surface-selection rules reduce the transition strength. For both ω_high_ and ω_low_ transitions, the diagonal width of the 2DIR peaks, which reflects their inhomogeneous bandwidth, is relatively broad (∼27 cm^–1^), indicating that the GSH ensemble is disordered.^38^

### Ag(I)– and Cu(I)–GSH Thiolates

In order to avoid a possible disorder of the molecular conformation associated with the presence of citrate molecules on the surface, all further experiments were conducted with bare AuNPs,^45^ which were prepared by the hydrogen reduction of the Au(III) oxide, as described in the Methods section. The addition of the Ag(I) cations into the AuNPs/GSH solution led to the formation of the Ag(I)–GSH aggregates on the surface of AuNPs. The 2DIR spectrum in Figure 5a features two main peaks that we previously found to characterize the β-sheet-like structure of the GSH on AgNPs:^23^ a strong peak at ω_β_1__ = 1626 cm^–1^, assigned to the excitons formed by the parallel beta-sheets, and a weak peak at ω_β_2__ = 1606 cm^–1^, assigned to β-sheets with even stronger interstrand hydrogen bonds, shorter distances between the amides, and a stronger interaction between their transition dipoles.^78^ Compared to the GSH aggregated on AgNPs, on AuNPs the diagonal width of the ω_β_1__ 2DIR peak is larger (∼23 vs ∼14 cm^–1^). Interestingly, regarding Ag(I)–GSH aggregates formed in bulk hydrogel in the absence of NPs, the 2DIR spectrum also features the same pattern of the ω_β_1__ and ω_β_2__ peaks, as shown in Figure 5b; however, the diagonal bandwidth of the ω_β_1__ peak is significantly smaller (∼13 cm^–1^, see diagonal slices of the 2DIR spectra in Figure 5c), matching that of the GSH on AgNPs. Therefore, the presence in a solution of residual Au(III) cations not reduced by hydrogen leads to a significant increase in the heterogeneity of the GSH aggregate structure.

Addition of Cu(I) cations to AuNP/GSH solution leads to a more complex situation because Cu(I) oxidizes in an aqueous solution.^79^ Nevertheless, GSH can reduce Cu(II) cations back to Cu(I), leading to the formation of the Cu(I)–GSH aggregates on AuNPs, when the acidity of the solution is maintained at ca. pH 3. We followed the procedure of ref (34) and adjusted the pH to the required value by adding NaOH. As shown in Figure 5d, also here the characteristic peaks appear at ω_β_1__ = 1630 and ω_β_2__ = 1606 cm^–1^, suggesting that the secondary structure of the peptide aggregates formed with Cu(I) is generally similar to that of Ag(I)–GSH aggregates. The diagonal width of the ω_β_1__ 2DIR peak (∼32 cm^–1^) is significantly larger than that of the Ag(I)–GSH aggregates on AuNPs, and its frequency is higher. On the other hand, regarding the Cu(I)–GSH aggregates in a bulk hydrogel, whose 2DIR spectrum is shown in Figure 5e, the ω_β_1__ frequency is the same as with Ag(I)–GSH, ω_β_1__ = 1626 cm^–1^, and the 2DIR peak is narrower (∼22 cm^–1^, see diagonal slices of the 2DIR spectra in Figure 5f). Assuming that all the M(I) thiolates have similar structures and that the chemical identity of the metal does not affect the range of the local order in the M(I)–thiolate aggregates, as predicted by theoretical modeling, these observations suggest that on AuNPs, the large heterogeneity is caused by the presence of the Au(III) and Cu(II) cations remaining in the solution.

Despite the increasing conformational heterogeneity of the GSH aggregates, the appearance of the ω_β_1__ and ω_β_2__ peaks in all samples at frequencies similar to the previously studied GSH on AgNPs suggests that the β-sheet-like structure is generally formed both on AuNPs and in the bulk hydrogels. To confirm that the corresponding peaks indeed represent vibrational excitons associated with the β-sheet conformation of the GSH peptide within the aggregate, we used peptides with the ^13^C stable isotope labels on the cysteine’s amide carbon. Labeling with ^13^C atom redshifts the transition frequency of the amide-I mode and allows one to evaluate the strength of the coupling between the transition dipoles on different peptide strands.^80^ Preparing a sample with a low concentration of the labeled atoms (typically ∼10% or less) allows one to obtain the individual transition frequency of the labeled amide in the conformation associated with the corresponding secondary structure of the peptide, whereas a fully labeled sample provides the frequency of the isotope-shifted exciton transition.^40,81^

2DIR spectra of the GSH aggregates with low-concentration and fully labeled ^13^C cysteine amides are shown in Figure 6 for Ag(I)–GSH on AuNPs, as well as for the Ag(I)–GSH and Cu(I)–GSH bulk hydrogel samples. Because of the relatively broad width of the transitions, ^13^C substitution does not shift the corresponding transitions in the low label concentration samples completely away from the unlabeled ones, and consequently, their peaks partially overlap. Nevertheless, the corresponding frequencies can be obtained from the 2DIR spectra owing to the spread of the spectral data in two dimensions. We obtained ω_C,10%_ ≈ 1612 cm^–1^ for Ag(I)–GSH on the AuNP sample, ω_C,10%_ ≈ 1606 cm^–1^ for the Ag(I)–GSH bulk hydrogel, and ω_C,6%_ ≈ 1609 cm^–1^ for the Cu(I)–GSH bulk hydrogel samples. For the fully labeled samples, we obtained ω_C,100%_ = 1597 cm^–1^, ω_C,100%_ = 1591 cm^–1^, and ω_C,100%_ = 1593 cm^–1^, respectively.

Despite the variation in the transition frequencies, relative intensities, and widths of the peaks observed in Figure 6, in all samples, the difference between the individual transition frequency obtained with the low label concentration sample and that of the excitonic transition obtained with the fully labeled sample is 15–16 cm^–1^, which is in agreement with the excitonic shift expected from the parallel β-sheet conformation of the peptide strands within the M(I)–GSH aggregate.^23^ At the same time, the mentioned variation in the details of the spectral peaks indicates that a variation exists in the microscopic structure of the corresponding β-sheet-like aggregates. These details, however, are beyond the sensitivity of the methodology used in the present work, and therefore, we do not discuss them here.

### Au(I)–GSH Thiolates

Similar to the case for the Ag(I) and Cu(I) cations, the addition of the Au(I) cations to the GSH sample leads to Au(I)–GSH aggregates formed as shells on the AuNP surface or as a bulk hydrogel in the absence of NPs. The Au(I) cations were generated in situ by tuning the pH via adding NaOH to AuNP/GSH or GSH-only solutions, respectively.^33^ 2DIR spectra of the Au(I)–GSH aggregates on AuNPs and in the bulk hydrogel, as shown in Figure 7a,b, feature similar peaks with transition frequencies of 1645 and 1627 cm^–1^ and a weak peak at 1600 cm^–1^. Isotope-dilution experiments with the ^13^C-labeled cysteine amide group revealed that the transition frequency of the individual amide-I vibrational mode obtained at a low label concentration, ω_C,10%_ ≈ 1604 cm^–1^, as shown in Figure 7d, undergoes only a small excitonic shift for the fully labeled sample, ω_C,100%_ ≈ 1600 cm^–1^, as shown in Figure 7e. This observation suggests that the fraction of the ordered content in the sample is small, the average number of strands participating in the β-sheet-like structures is low, and the peptide conformation of this hydrogel is mostly disordered.

## Conclusions

To summarize, we demonstrated a method of coating the surface of noble metal NPs (Au and Ag) with a few-nanometer-thick hydrogel shell composed of GSH aggregates that have a parallel β-sheet-like secondary structure. The formation of the β-sheets is induced by M(I) thiolates, where M(I) stands for the coinage metals Ag, Cu, and Au. Theoretical analysis of the molecular conformations suggests that oligomers with a parallel strand configuration aggregate via kinetic pathways. Calculated M(I)–M(I) distances in a simplified model of the M(I)–GSH β-sheet of ∼4.5 Å suggest that a seed layer can, in principle, coordinate at the metal centers on the surface, where the interatomic distances are of a comparable length. However, for the M(I)–GSH aggregates in bulk hydrogels having an analogous molecular conformation, the M(I)–M(I) distances should be compared to those found in molecular complexes with homoatomic metallophilic close-shell d^10^–d^10^ interactions.^82^ These interactions are typically shorter than those obtained in our calculations, about 3.0–3.5 Å. Counterintuitively, the M(I)–M(I) interactions in such metallophilic complexes are thought to be weakly repulsive,^83^ such that the complex is kept together by the attraction of the van der Waals forces between the ligands.^84^ Therefore, these considerations indicate that molecular conformations within the M(I)–GSH aggregates are predominantly governed by the hydrogen bond and zwitterion interactions between the peptide strands.

2DIR spectroscopy indicates that the β-sheet-like structure of the M(I)–GSH aggregates on NPs is similar to that in the bulk hydrogels, whereas the theoretical analysis suggests that the β-sheet-like structure has similar conformations for all of the M(I) thiolates, regardless of the chemical identity of the coinage metal. Experimental results show that conformational disorder within the β-sheets increases with the increase in the highest possible oxidation number of the coinage metal. The M(II) and M(III) cations, present in the sample solution, can be reduced by the GSH to their M(I) oxidation state, producing oxidized GSSG dimers, which can admix into the aggregate and perturb its order. We tested the possibility that the fraction of the ordered structure in the aggregates depends on the amount of the oxidized GSSG dimers added to the sample and found that this is not the case - the dimers are removed from the sample at the stage where the solvent is changed from H_2_O to D_2_O, required for the infrared spectroscopy (see the Methods section), whereas the aggregate’s order is not affected.

There is great interest in developing biomolecule-functionalized nanomaterials for various applications, which include nanoparticles functionalized with self-assembled monolayers of peptides, multilayer self-assembled molecular shells, and protein corona. In the current study, we established conditions when a thiolate-based shell of controlled thickness could be formed on NPs while avoiding the formation of a bulk hydrogel. The noble metal NPs with a peptide hydrogel shell present an unusual combination of materials with different properties: the metal core possesses tunable plasmonic resonances, and the entire shell has a well-defined ordered structure despite that its thickness is significantly larger than that of the ordered self-assembled monolayers, whereas its hydrogel nature opens opportunities to employ the soft peptide framework for loading and scaffolding. Therefore, we expect that NPs with ordered hydrogel shells will be useful additions to the nanomaterial toolbox. Moreover, we anticipate that it will be possible to fabricate such bionanogel coatings not only on noble metals but also on arbitrary surfaces, where the seed layer of the GSH peptide can be deposited.

## Supporting Information Available

TEM imaging and description of the theoretical models used to interpret electrophoretic measurements (PDF)
