# Supplementary material for: Noble Metal Nanoparticles with Nanogel Coatings: Coinage Metal Thiolate-Stabilized Glutathione Hydrogel Shells
Source: J Phys Chem C Nanomater Interfaces. 2024 Feb 14;128(8):3438–48. doi: 10.1021/acs.jpcc.4c00433 (PMC10911076; doi:10.1021/acs.jpcc.4c00433)
Supplement: Supplementary file 1 — jp4c00433_si_001.pdf [file jp4c00433_si_001.pdf]

Noble Metal Nanoparticles with Nanogel Coatings: Coinage Metal Thiolate-Stabilized Glutathione Hydrogel Shells

Arghyadeep Basu,[1] Iogann Tolbatov,[2] Alessandro Marrone,[3] Alexander Vaskevich,[4] and Lev Chuntonov[1,*]

[1]Schulich Faculty of Chemistry and Solid-State Institute, Technion – Israel Institute of Technology, Haifa 3200003, Israel; [2]Department of Physics and Astronomy, University of Padova, via F. Marzolo 8, 35131, Padova, Italy; Institute of Chemical Research of Catalonia (ICIQ), Barcelona Institute of Science and Technology, Av. Països Catalans 16, 43007 Tarragona, Spain; [3]Dipartimento di Farmacia, Università degli Studi "G. D'Annunzio" Chieti-Pescara, Via dei Vestini, 66100 Chieti, Italy; [4]Department of Molecular Chemistry and Materials Science, Weizmann Institute of Science, Rehovot 7610001, Israel

Supporting Information

TEM imaging

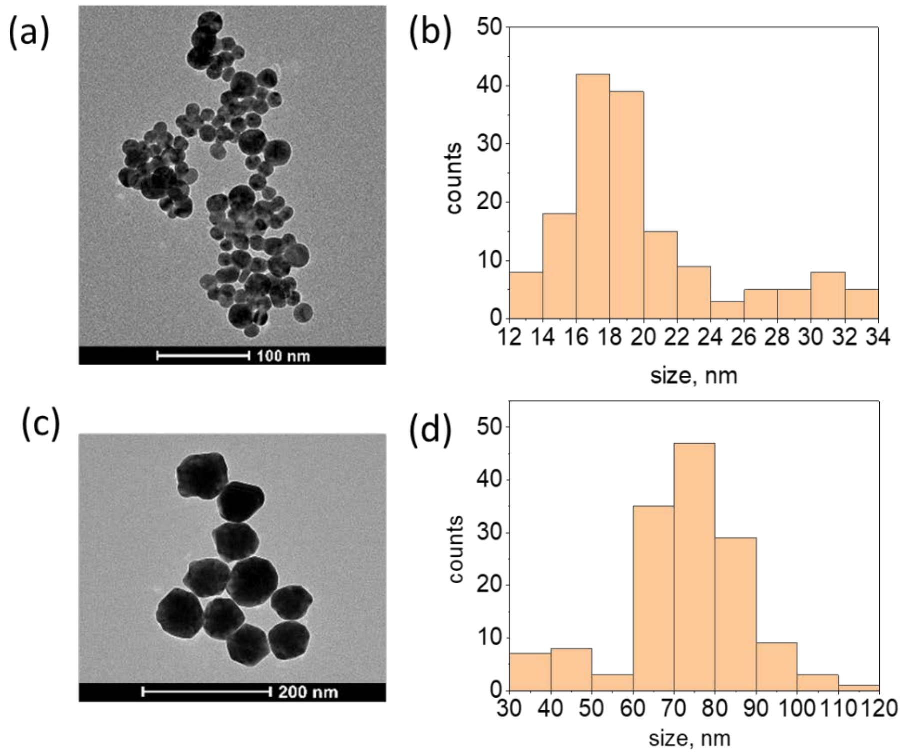

Figure S1. Transmission electron microscopy of AuNPs. Micro-images of citrate-capped NPs (a) and the corresponding statistical analysis (b). The mean size is 20 nm and the standard deviation is 5 nm. Micro-images of bare AuNPs prepared by the hydrogen reduction method (c) and the corresponding statistical analysis (d). The mean size is 73 nm and the standard deviation is 15 nm.

Electrophoretic mobility

Results of the electrophoretic mobility measurements of bare AuNPs (Figure 2c of the main text) were analyzed following the work of Ohshima and co-workers.[1,2] For the case of rigid NPs with constant surface charge density, electrophoretic mobility is given by

$$\mu = \frac{2\varepsilon_r\varepsilon_0\zeta}{3\eta}\left(1+\frac{1}{2\left[1+\frac{2.5}{\kappa a(1+2e^{-\kappa a})}\right]^3}\right) - \frac{2\varepsilon_r\varepsilon_0}{3\eta}\left(\frac{ze\zeta}{\kappa T}\right)^2 \times$$

$$\left(\frac{\kappa a(\kappa a+1.3\exp(-0.18\kappa a)+2.5)}{2(\kappa a+1.2\exp(-7.4\kappa a)+4.8)^3}+\left(\frac{m_+ + m_-}{2}\right)\frac{9\kappa a(\kappa a+5.2\exp(-3.9\kappa a)+5.6)}{8(\kappa a-1.55\exp(-0.32\kappa a)+6.02)^3}\right), \quad \text{(S1)}$$

where ε_r is the relative permittivity of the solvent, ε_0 is the vacuum permittivity, ζ is the zeta-potential, η is the viscosity and z is the valence of the symmetrical electrolyte solution with the bulk number concentration n^∞, e is the electron charge, k_B is the Boltzmann constant, T is the temperature, a is the radius of the spherical NP, κ is the Debye parameter, $\kappa = \left(\frac{2n^{\infty 2}z^2e^2}{\varepsilon_r\varepsilon_0 k_\mathrm{B}T}\right)^{1/2}$, $m_\pm$ are dimensionless ionic drag coefficients $m_\pm = \frac{2\varepsilon_r\varepsilon_0 k_B T}{3\eta z^2 e^2}\lambda_\pm$, $\lambda_\pm$ are ion drag coefficients $\lambda_\pm = \frac{\mathrm{N_A}\, e^2 z}{\Lambda^0_\pm}$, N_A is the Avogadro's number, $\Lambda^0_\pm$ are the ion conductances at infinite dilution.

The surface charge density is related to the potential via

$$\sigma = \frac{2\varepsilon_r\varepsilon_0\kappa k_B T}{ze}\sin\left(\frac{ze\zeta}{2k_BT}\right)\left[1+\frac{1}{\kappa a}\frac{2}{\cosh^2\left(\frac{ze\zeta}{4kT}\right)}+\frac{1}{(\kappa \mathrm{a})^2}\frac{8\ln\left[\cos\left(\frac{ze\zeta}{4kT}\right)\right]}{\sinh^2\left(\frac{ze\zeta}{2kT}\right)}\right]^{\frac{1}{2}}, \quad \text{(S2)}$$

which can be approximately re-expressed as

$$\zeta \approx \frac{2k_BT}{ze}\sinh^{-1}\frac{ze\sigma}{2\varepsilon_r\varepsilon_0\kappa k_B T}. \quad \text{(S3)}$$

Equation S1 was used together with the equation S3 to fit the experimental data in Figure 2c.

Results of the electrophoretic mobility measurements of AuNPs coated with Ag(I)-GSH thiolate shell (Figure 2d of the main text) were analyzed following the work of Ohshima and co-

workers.[3,4] For the case of NPs with rigid core and soft shell permeable by the solvent electrolyte, electrophoretic mobility is given by

$$\mu = \frac{\varepsilon_r \varepsilon_0}{\eta} \frac{\psi_0/\kappa_m + \psi_{\mathrm{DON}}/\lambda}{1/\kappa_m + 1/\lambda} + \frac{ZeN}{\eta\lambda^2}, \tag{S4}$$

where ψ_{DON} is the Donnan potential, ψ_0 is the surface potential of the spherical NP, Z is the valence and N is the concentration of the fixed ions within the soft permeable NP shell, and λ^{-1} is the softness parameter and κ_m is the modified Debye parameter. The ψ_{DON}, ψ_0, and κ_m are given by the following expressions:

$$\psi_{\mathrm{DON}} = \frac{k_B T}{ze} ln \left[\frac{ZN}{2zn^{\infty}} + \left(\left(\frac{ZN}{2zn^{\infty}} \right)^2 + 1 \right)^{1/2} \right], \tag{S5}$$

$$\psi_0 = \psi_{\mathrm{DON}} + \frac{2n^{\infty} k_B T}{ZeN} \left[1 - \left(\left(\frac{ZN}{2zn^{\infty}} \right)^2 + 1 \right)^{1/2} \right], \tag{S6}$$

and

$$\kappa_m = k \left[1 + \left(\frac{ZN}{2zn^{\infty}} \right)^2 \right]^{\frac{1}{4}}. \tag{S7}$$

Equations S4-S7 were used to fit the experimental data in Figure 2d.

References

(1) Agnihotri, S. M.; Ohshima, H.; Terada, H.; Tomoda, K.; Makino, K. Electrophoretic Mobility of Colloidal Gold Particles in Electrolyte Solutions. *Langmuir* **2009,** *25* (8), 4804.

(2) Makino, K.; Ohshima, H. Electrophoretic Mobility of a Colloidal Particle with Constant Surface Charge Density. *Langmuir* **2010,** *26* (23), 18016.

(3) Makino, K.; Ohshima, H. Soft particle analysis of electrokinetics of biological cells and their model systems. *Sci .Technol. Adv. Mater.* **2011,** *12* (2), 023001.

(4) Ohshima, H. Theory of electrostatics and electrokinetics of soft particles. *Sci .Technol. Adv. Mater.* **2009,** *10* (6), 063001.
